# Efficacy evaluation and mechanistic investigation of transcutaneous auricular vagus nerve stimulation in enhancing the therapeutic effects of semaglutide and reducing its gastrointestinal side effects: a randomized controlled trial protocol

**DOI:** 10.3389/fneur.2025.1653900

**Published:** 2025-12-05

**Authors:** Xinyi Tian, Lu Zhang, Kaiqi Zhang, Xiaolei Ge, Zhengrong Luo, Youwen Zhang, Ge Wang, Xu Zhai

**Affiliations:** 1 School of Acupuncture-Moxibustion and Tuina, Shandong University of Traditional Chinese Medicine, Jinan, China; 2 Institute of Chinese Medical History and Literatures, China Academy of Chinese Medical Sciences, Beijing, China; 3 Wangjing Hospital, China Academy of Chinese Medical Sciences, Beijing, China; 4 Graduate School, China Academy of Chinese Medical Sciences, Beijing, China

**Keywords:** transcutaneous auricular vagus nerve stimulation, semaglutide, randomized controlled trial, side effects, type 2 diabetes mellitus, gastrointestinal side effects

## Abstract

**Background:**

Type 2 diabetes mellitus (T2DM) is a chronic disorder with serious complications. Semaglutide, a GLP-1 receptor agonist, improves glycemic control and weight but is limited by gastrointestinal (GI) side effects, notably nausea, vomiting, and diarrhea, which impair adherence. Transcutaneous auricular vagus nerve stimulation (taVNS), a non-invasive therapy, shows potential in alleviating gastrointestinal symptoms, yet its role in mitigating semaglutide-related side effects and enhancing drug efficacy remains unexplored.

**Methods:**

A total of 60 T2DM participants experiencing semaglutide-induced GI side effects will be randomly assigned to a control or intervention group. The control group will receive semaglutide (0.25 mg weekly) plus sham-taVNS, with electrodes attached to the bilateral auricular scapha at 4/20 Hz for 30 min, twice daily. The intervention group will receive semaglutide plus active taVNS, with electrodes on the bilateral cymba and cavum conchae using the same stimulation parameters. Treatment will span two 6-week cycles (12 weeks total). The primary outcome is the Rhodes Index of Nausea, Vomiting, and Retching. Secondary outcomes include gastric electromyography, serological markers, Visual Analogue Scale, and the Simplified Nutritional Appetite Questionnaire. Adverse events will be monitored, and assessments will occur at baseline, week 6, end of treatment, and follow-up.

**Discussion:**

This study aims to determine whether taVNS can alleviate semaglutide-induced GI side effects and enhance the therapeutic efficacy of semaglutide.

**Conclusion:**

This study protocol has a randomized, sham-controlled design with rigorous internal validity. Limitations include the potential placebo effects, single-center setting, and small sample size. We expect taVNS to alleviate semaglutide-induced GI side effects and potentially enhance its therapeutic efficacy. In conclusion, this trial will provide preliminary evidence on the safety and effectiveness of taVNS as an supplementary therapy to semaglutide in T2DM, informing the design of future larger-scale studies.

**Clinical trial registration:**

http://itmctr.ccebtcm.org.cn/en-US, identifier ITMCTR2025001226.

## 1 Introduction

Type 2 diabetes mellitus (T2DM) is a chronic metabolic disorder characterized by insulin resistance and impaired insulin secretion. Since its approval, semaglutide—a novel glucagon-like peptide-1 (GLP-1) receptor agonist—has attracted considerable attention (1). It significantly improves glycemic control by enhancing insulin secretion, suppressing glucagon release, and delaying gastric emptying. Additionally, semaglutide is convenient to use and associated with a low risk of hypoglycemia and cardiovascular adverse events (2). However, its clinical utility is somewhat limited by GI side effects such as nausea and vomiting, which are primarily attributed to its direct action on the GI tract and its ability to delay gastric emptying during the first postprandial hour (3). Signal detection based on reports collected in the FDA Adverse Event Reporting System (FAERS) database from December 2017 to December 2020 identified the three most frequently reported adverse drug events (ADEs) related to semaglutide as nausea, vomiting, and diarrhea. Approximately 20–35% of patients discontinued semaglutide due to GI reactions, which significantly affect treatment adherence and quality of life (4), thereby limiting its broader clinical use. Current strategies for managing nausea and vomiting include both pharmacological interventions (5) and non-pharmacological approaches, such as acupuncture (6), electroacupuncture (7), tuina (8), transcutaneous auricular vagus nerve stimulation (taVNS) (9), and auricular point pressing therapy (10).

Serotonin (5-HT) antagonists (e.g., ondansetron) and dopamine (DA) antagonists (e.g., metoclopramide) are first-line antiemetics, blocking serotonin or dopamine signaling to relieve nausea and vomiting (11, 12). Corticosteroids (e.g., dexamethasone) further reduce gastrointestinal inflammation (13). However, these medications are associated with varying degrees of adverse effects. For instance, common side effects of ondansetron include headache and constipation (14), metoclopramide may induce extrapyramidal symptoms such as tremors and muscle rigidity (15), and dexamethasone is known to elevate blood glucose levels and blood pressure (16). These adverse effects make them less favorable for use in T2DM patients.

taVNS is a novel, non-invasive, and low-cost neuromodulation method derived from Traditional Chinese Medicine auricular acupuncture. By electrically stimulating the auricular branch of the vagus nerve (ABVN), taVNS regulates glucose metabolism, gastrointestinal motility, obesity, and immune responses via vagal pathways (17, 18). taVNS delivers electrical stimulation to the auricular branch of the vagus nerve, activating central nuclei and vagal pathways. It modulates autonomic balance, influences peripheral organ functions, and provides VNS-like therapeutic effects across central and peripheral systems (19). A recent 2024 preclinical study demonstrated that taVNS could improve gastric motility and visceral hypersensitivity in a rodent model of functional dyspepsia (FD) by restoring duodenal immune homeostasis (20). The study found that taVNS activated the NTS and DMN via vagovagal reflexes, leading to enhanced GI motility and reduced low-grade duodenal inflammation, supporting its potential as a first-line non-pharmacological therapy for FD. Due to its non-invasiveness, portability, efficacy, and minimal side effects, taVNS is widely favored by patients. Clinical studies have shown that taVNS can alleviate constipation and abdominal pain in patients with irritable bowel syndrome with constipation (IBS-C) (21), and improve gastric motility and hypersensitivity through vagus nerve-mediated anti-inflammatory mechanisms (22, 23). Moreover, taVNS has been shown to reduce blood glucose levels in T2DM patients by enhancing vagal efferent activity and stimulating GLP-1 secretion (24), and to improve glucose tolerance in individuals with impaired glucose regulation (25). Previous clinical trials have primarily focused on using taVNS to treat isolated GI conditions (26–28) and postoperative nausea and vomiting (29–31). However, to date, no clinical studies have specifically evaluated the effect of taVNS in alleviating GI side effects, such as nausea and vomiting, induced by semaglutide, nor its impact on the therapeutic efficacy of semaglutide. Therefore, the present study aims to evaluate the clinical efficacy and underlying mechanisms of taVNS in mitigating semaglutide-induced GI side effects, as well as its potential role in enhancing the pharmacological effect of semaglutide. This investigation further explores the prospects of taVNS as a non-pharmacological intervention for T2DM and related endocrine disorders.

## 2 Methods

### 2.1 Study design

**Figure 1:**
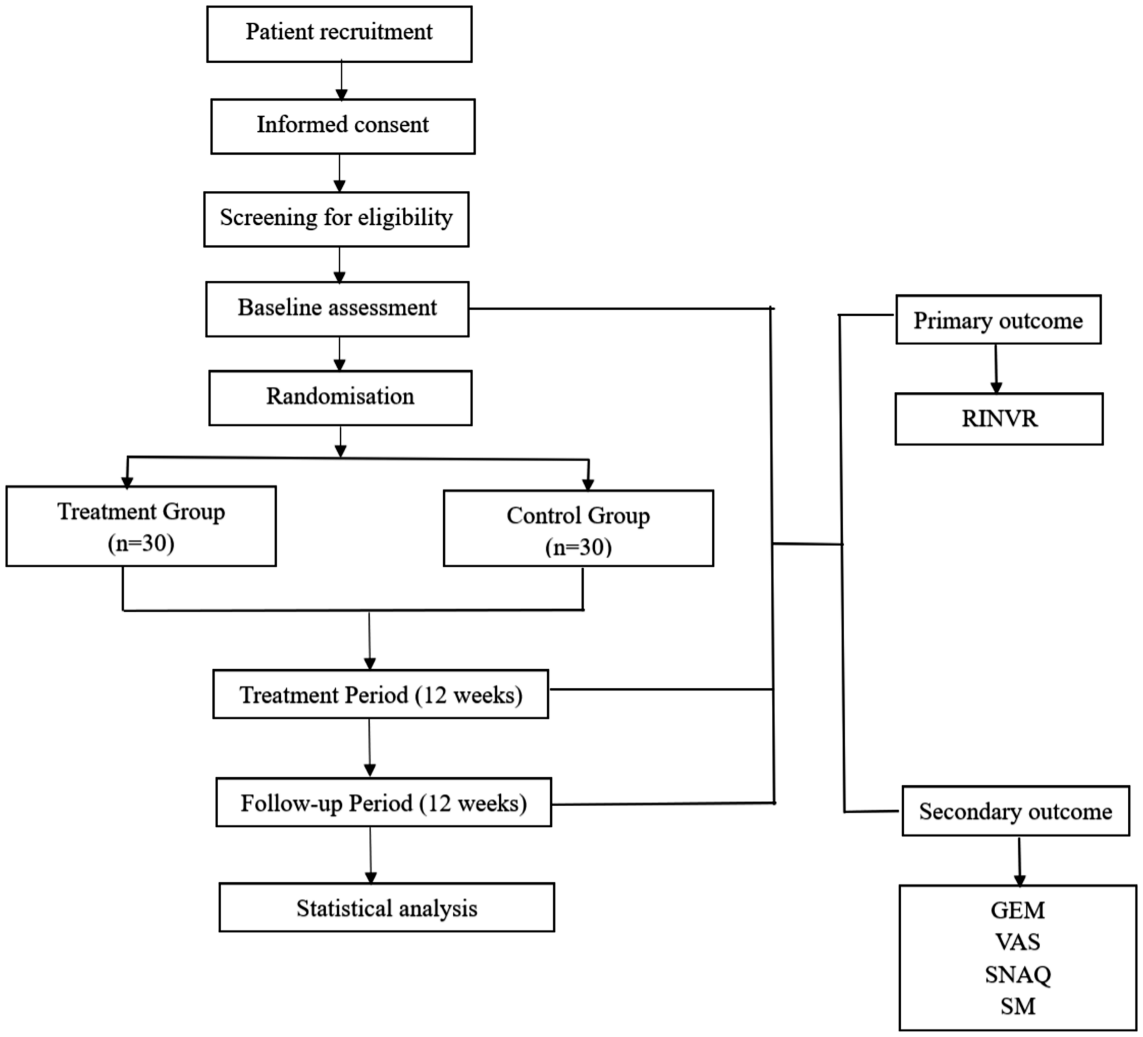
Schematic diagram of the study protocol. RINVR: Rhodes Index of Nausea, Vomiting and Retching; GEM: Gastric Electromyography; VAS: Visual analogue scale; SNAQ: Simplified Nutritional Appetite Questionnaire Short Form. SM: Serological Markers.

This study is designed as a single-center, randomized, sham-controlled trial (RCT) to evaluate the efficacy and safety of taVNS in alleviating GI side effects such as nausea and vomiting induced by semaglutide, as well as its potential impact on the drug’s therapeutic efficacy. The study protocol has been developed in accordance with the SPIRIT (Standard Protocol Items: Recommendations for Interventional Trials) checklist. A total of 60 participants with T2DM will be randomly assigned in a 1:1 ratio to either the intervention group or the control group (Figure 1). The institutional ethics committee has granted approval for this protocol. To ensure ongoing ethical compliance and participant safety, quarterly audits will be performed; these audits will evaluate the necessity for premature study termination. All trial procedures will strictly adhere to the principles outlined in the Declaration of Helsinki (2013 revision). Prior to enrollment, every participant will provide documented informed consent.

### 2.2 Recruitment

Recruitment will utilize multiple strategies: online advertisements disseminated through the hospital’s official website and the WeChat social media platform (China), alongside physical flyers placed within outpatient clinic areas.

Participants will be recruited from the Department of Acupuncture at Wangjing Hospital, China Academy of Chinese Medical Sciences, located in Chaoyang District, Beijing. Enrollment will begin in November 2025 and is expected to be completed by May 2026. Using a 1:1 randomization scheme, consenting eligible participants will be assigned to receive twelve weeks of either intervention group or control group. The research team will recruit eligible participants based strictly on predefined inclusion and exclusion criteria. All participant data will be maintained under the exclusive custodianship of the Data Monitoring Committee (DMC). Access to study data and participant identities is restricted to authorized study personnel, including members of the DMC, principal investigators, and designated research staff involved in data collection and analysis.

#### 2.2.1 Diagnostic criteria

The diagnosis of diabetes will follow the Chinese Guidelines for the Prevention and Treatment of Diabetes (2024 edition) and the diagnostic criteria of the World Health Organization (WHO) (32). A diagnosis can be established if any one of the following conditions is met:

(1) Fasting plasma glucose (FPG) ≥ 7.0 mmol/L (126 mg/dL); Fasting is defined as no caloric intake for at least 8 h.
(2) 2-h plasma glucose (2 h-PG) ≥ 11.1 mmol/L (200 mg/dL) during an oral glucose tolerance test (OGTT); Blood glucose is measured 2 h after ingestion of a solution containing 75 g of anhydrous glucose.
(3) Glycated hemoglobin (HbA1c) ≥ 6.5%; Must be measured using internationally standardized methods (e.g., NGSP/DCCT standardized).
(4) Random plasma glucose ≥ 11.1 mmol/L (200 mg/dL) in the presence of typical symptoms; Typical symptoms include polydipsia, polyuria, unexplained weight loss, or blurred vision.

#### 2.2.2 Inclusion criteria

(1) Age between 18 and 70 years.
(2) Meets diagnostic criteria for T2DM: FPG ≥ 126 mg/dL (7.0 mmol/L), or HbA1c ≥ 6.5%, or 2 h-PG ≥ 200 mg/dL (11.1 mmol/L) after glucose loading.
(3) Experiences GI discomfort symptoms such as nausea or vomiting within one month. After semaglutide injection, with a RINVR score ≥ 20 and ≤ 32.
(4) Abnormal gastric electromyography (GEM) findings after a test meal (egg sandwich + 200 mL water), characterized by a baseline rhythm frequency < 2.4 cpm, no significant increase in power spectral density, and decreased frequency and amplitude of tachygastria.
(5) Provides written informed consent.

#### 2.2.3 Exclusion criteria

(1) Participants with severe cardiac, cerebrovascular, hepatic, hematologic, or other life-threatening conditions.
(2) Participants with hepatic or renal insufficiency, or liver cirrhosis.
(3) Participants with high fever or infectious diseases.
(4) Pregnant or lactating women.
(5) Participants with psychiatric disorders or who are unable to comply with the treatment protocol.
(6) Participants with cardiac pacemakers, known or suspected allergy to the treatment used in this study.
(7) Participants with acute pancreatitis, diabetic retinopathy, or thyroid disorders.
(8) Participants currently taking prokinetic drugs or other antiemetics (e.g., ondansetron, metoclopramide, dexamethasone).
(9) Participants with GI disorders that may cause nausea and vomiting (e.g., diabetic gastroparesis).
(10) Participants who have participated in other clinical trials within the past 3 months and may be at risk of interference with this study.

### 2.3 Sample size

This study is designed as an exploratory pilot trial. As no previous studies have directly examined the effect of taVNS on the efficacy and GI side effects of semaglutide, a formal power-based sample size calculation was not feasible. Nevertheless, we recognize the importance of providing a rationale for the chosen sample size. According to phase III clinical trials of semaglutide, GI adverse events such as nausea, vomiting, and diarrhea occur in approximately 10–25% of patients (33). In addition, network meta-analyses have shown that GLP-1 receptor agonists increase the overall risk of GI adverse events with an odds ratio of about 4.5 compared to placebo (34).

Considering a continuous outcome measure (RINVR score) and assuming a moderate effect size (Cohen’s d ≈ 0.5), a sample size of 25–30 participants per group would be expected to provide approximately 70–80% power, allowing for a preliminary evaluation of efficacy trends at a two-sided *α* of 0.05. Taking into account an estimated 10% dropout rate, we plan to recruit 60 participants in total, allocated 1:1 to the experimental and control groups, which is considered sufficient to assess feasibility, safety, and preliminary efficacy, and to inform the sample size calculation of future large-scale randomized controlled trials.

### 2.4 Randomization

Block randomization will be employed to ensure balance in prognostic factors across intervention groups. Randomization will follow a basic procedure. The allocation sequence, produced using SPSS software (v24.0), will assign participants equally (1,1) to the intervention group or the control group. For allocation concealment, this list will be securely enclosed in opaque envelopes. Blinded circulating nurse will distribute these envelopes to enrolled participants.

### 2.5 Blinding

Prior to participant enrollment, a blinded circulating nurse will open the allocation envelopes to determine each participant’s group assignment. Each envelope contains the allocation information for only one individual participant, ensuring that group assignments remain concealed until the time of enrollment. After verifying the allocation details, the envelopes will be resealed, and this procedure will be conducted without disclosing any information to the participants. To ensure effective blinding, participants will be informed during the pre-enrollment consultation about potential sensory experiences during treatment, including tingling, pricking sensations, temperature, and vibration. It will be clearly emphasized that individual perception of stimulation may vary, and changes in intensity or location should not be used to assess treatment efficacy. All stimulation devices will have a standardized appearance, with differences limited to the electrical output location, thereby further preserving blinding integrity. Outcome assessors, data analysts, and investigators will remain blinded to group allocation. Prior to study initiation, outcome assessors will undergo rigorous training on evaluation procedures and standards to minimize subjective bias in outcome assessment.

### 2.6 Intervention measures

All operators (acupuncturists) involved in this study have over two years of clinical experience and hold valid licenses as TCM practitioners. An electroacupuncture device (Model 219, Huatuo, SDZ-IIB, Suzhou, China) with specialized silicone ear loops will be used. Based on traditional Chinese auricular points theory and the mechanism by which semaglutide induces GI adverse effects, including delayed gastric emptying and inhibition of the hypothalamic appetite center, the auricular points CO_2_ and CO_6_ were selected as stimulation sites for taVNS. These points are located in the crus of the cymba conchae and the cavum conchae, respectively, thereby simultaneously stimulating the auricular branch of the vagus nerve within the conchal region during the intervention.

On the day of enrollment, each participant will be trained on the use of the device and instructed on relevant precautions, ensuring that they are capable of performing self-administered treatment at home. Treatment will begin on the second day after randomization. Both groups will receive bilateral ear stimulation for 30 min per session, twice daily. In accordance with the Declaration of Helsinki, if a participant experiences severe GI side effects from semaglutide that interfere with daily life, temporary pharmacological intervention is permitted, and the drug name and dosage will be reported to the responsible physician. Medication history will be systematically recorded in the case report forms.

#### 2.6.1 Control group

Participants in the control group will receive semaglutide injections and sham taVNS intervention: Semaglutide injection (0.25 mg, 1.34 mg/mL, Ozempic, Novo Nordisk) will be administered once weekly for 12 weeks, totaling 12 doses.

**Figure 2:**
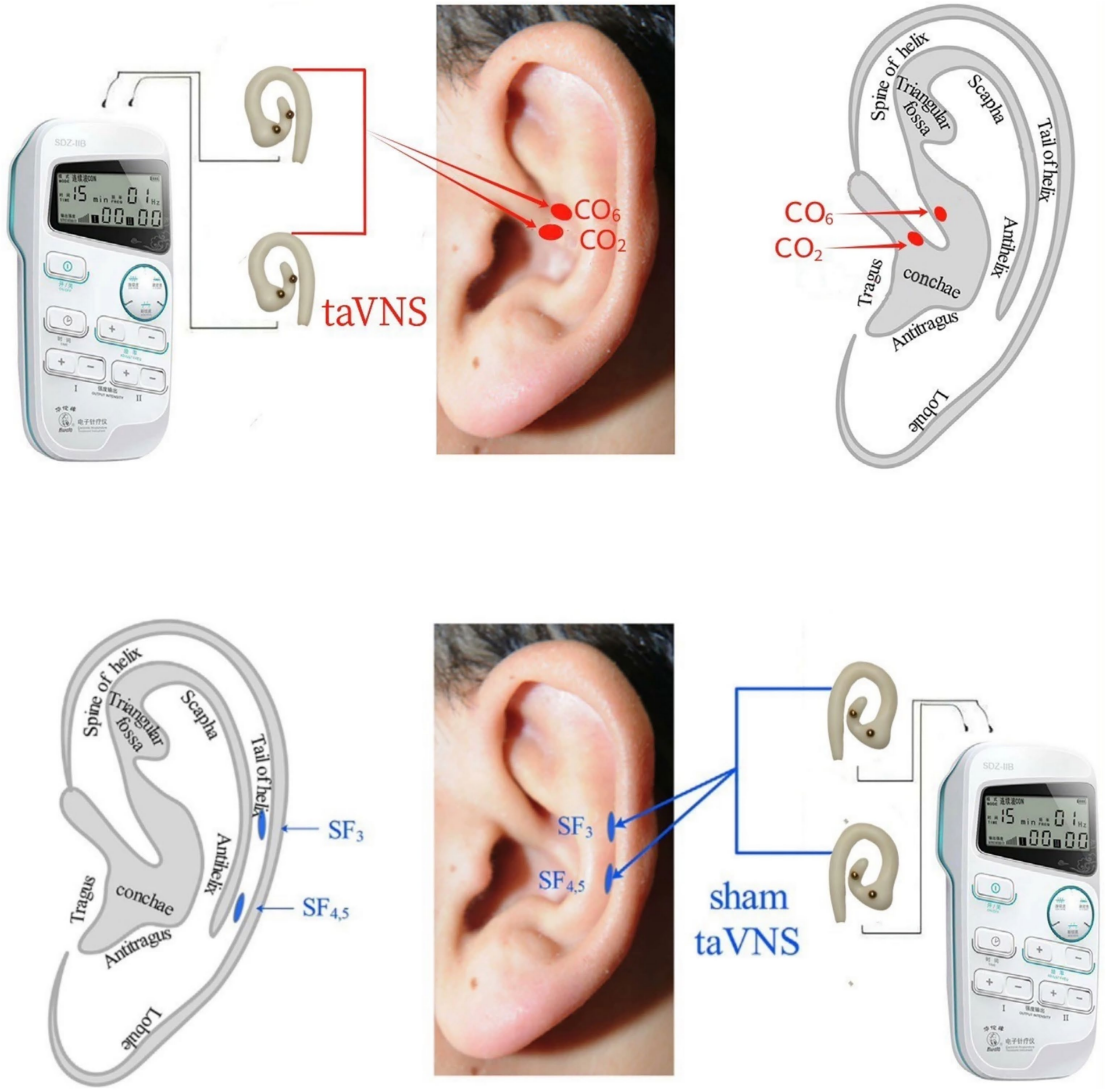
taVNS and sham-taVNS. taVNS: transcutaneous auricular vagus nerve stimulation. CO_2_ (Esophagus): Located at the lower one-third of the crus of the helix, corresponding to concha region 2. CO_6_ (Small Intestine): Located at the middle one-third between the crus of the helix and part of the helix along the AB line, corresponding to concha region 6. SF_3_, SF_4.5_: Sham points (auricular scapha): Two points located in the auricular scapha at the same horizontal level as CO_2_ and CO_6_, used as sham stimulation sites.

Sham taVNS: After cleaning the auricular skin with alcohol, ear loops will be attached to the bilateral auricular scapha (these areas are mainly innervated by the great auricular and auriculotemporal nerves rather than the auricular branch of the vagus nerve) and connected to the stimulator using a 4/20 Hz sparse-dense waveform (Figure 2). Treatment will be administered twice daily for 30 min per session over 12 weeks.

#### 2.6.2 Intervention group

Participants in the intervention group will receive semaglutide injections combined with active taVNS: Semaglutide injection (0.25 mg, 1.34 mg/mL, Ozempic, Novo Nordisk) will be administered once weekly for 12 weeks, totaling 12 doses.

taVNS: After cleaning the auricular skin with alcohol, ear loops will be attached to the bilateral cavum conchae and cavum conchae (a region considered to have rich auricular vagus nerve innervation) and connected to the stimulator using a 4/20 Hz sparse-dense waveform (Figure 2). Treatment will be administered twice daily for 30 min per session over 12 weeks.

### 2.7 Outcome measures

#### 2.7.1 Primary outcome measure

##### 2.7.1.1 The Rhodes index of nausea, vomiting, and retching (RINVR)

The RINVR consists of 8 items, each scored by the patient based on subjective experience using a Likert scale ranging from 0 to 4 (35). The total score ranges from 0 to 32. The scale evaluates the following three domains:

1. Nausea-related items

Frequency of nausea (number of times nausea is experienced per day); Duration of nausea (length of each episode); Severity of nausea (degree of discomfort caused by nausea).

2. Vomiting-related items

Frequency of vomiting (number of vomiting episodes per day); Amount of vomitus (volume of expelled contents per episode); Severity of vomiting (degree of discomfort caused by vomiting).

3. Retching-related items

Frequency of retching (number of retching episodes per day; retching refers to vomiting reflex without expulsion); Severity of retching (degree of discomfort caused by retching).

#### 2.7.2 Secondary outcome measures

##### 2.7.2.1 Serological markers (SM)

Serological markers will be monitored using liquid-phase suspension chip technology. A 2 mL fasting venous blood sample will be collected from each participant in the early morning. Within 30 min of collection, the samples will be aliquoted and cryopreserved according to standard procedures. The following serological markers will be assessed: fasting insulin, C-peptide, fasting glucagon, fasting plasma glucose, 2-h postprandial glucose, glycated albumin, leptin, adiponectin, lipid profile (triglycerides, total cholesterol, low-density lipoprotein cholesterol, high-density lipoprotein cholesterol), liver function, renal function, gastrin-17, motilin, 5-HT, DA, HbA1c, and cholecystokinin. Contingency plans will be in place for unexpected events; for example, high-sugar foods will be prepared in advance in case of hypoglycemia.

##### 2.7.2.2 Gastric electromyography (GEM)

Under normal postprandial conditions, gastric electrogastrography displays a regular pattern of electrical activity that corresponds to normal gastric contractions and relaxations (36). Gastric hypomotility may lead to abnormalities in these patterns, such as alterations in frequency and amplitude.

The normal postprandial baseline rhythm typically maintains a frequency of approximately 3 cycles per minute (cpm), with a normal range of 2.4–3.7 cpm. Within this frequency range, an increase in power spectral density is usually observed, accompanied by enhanced spike potential activity.

In cases of delayed gastric emptying, the baseline frequency often falls below the normal range (commonly <2.4 cpm), with no significant increase in power spectral density. Spike potentials may decrease in both frequency and amplitude, and abnormal rhythms may present, including irregular frequencies, inconsistent wave amplitudes, or desynchronization of rhythms between different gastric regions.

##### 2.7.2.3 Visual analogue scale (VAS)

The Visual Analogue Scale (VAS) is widely used in China (37). It consists of a 10-centimeter horizontal line marked with a continuum of 10 value points. The two endpoints represent extremes of symptom severity, with “0” indicating no symptoms of nausea or vomiting, and “10” representing the most severe and unbearable nausea and vomiting.

**Table 1:** 

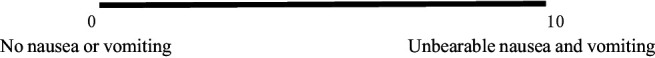

##### 2.7.2.4 Simplified nutritional appetite questionnaire short form (SNAQ)

SNAQ is a tool used to assess an individual’s appetite and the risk of inadequate nutritional intake (38). It is commonly applied in older adult populations or among participants to identify early signs of malnutrition. The SNAQ consists of four brief questions addressing appetite, satiety, taste perception, and meal frequency:

1. Appetite level

Very poor-5 points; Poor-4 points; average-3 points; Good-2 points; Very good-1 point.

2. When do you feel full while eating

After just a few bites-5 points; after eating one-third of a meal-4 points; after eating half of a meal-3 points; near finishing a meal-2 points; Rarely feel full-1 point.

3. Taste perception of food

Very poor-5 points; Poor-4 points; average-3 points; Good-2 points; very good-1 point.

4. Normal number of meals per day

Less than one meal/day-5 points; one meal/day-4 points; two meals/day-3 points; three meals/day-2 points; more than three meals/day-1 point.

#### 2.7.3 Outcome measurements

The Rhodes Index of Nausea, Vomiting, and Retching (RINVR), Simplified Nutritional Appetite Questionnaire (SNAQ), and Visual Analogue Scale (VAS) will be recorded at baseline (pre-treatment), at six weeks, and at twelve weeks post-treatment. Permission to use the RINVR, SNAQ, and VAS was obtained from the respective copyright holders.

**Figure 3:**
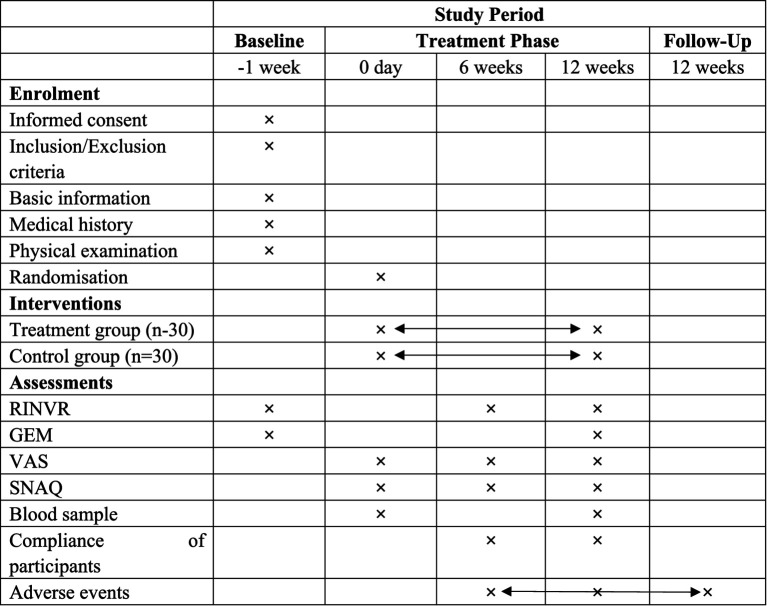
Outcome measures collection schedule. ×: the moment of enrolment, intervention or assessments. Double-headed arrows: the whole phase of intervention or adverse events assessment. RINVR: Rhodes Index of Nausea, Vomiting and Retching; GEM: Gastric Electromyography; VAS: Visual analogue scale; SNAQ: Simplified Nutritional Appetite Questionnaire Short Form.

Serological Markers—including fasting insulin, C-peptide, fasting glucagon, fasting plasma glucose, 2-h postprandial glucose, glycated albumin, leptin, adiponectin, lipid profile (triglycerides, total cholesterol, low-density lipoprotein cholesterol, high-density lipoprotein cholesterol), liver function, renal function, gastrin-17, motilin, 5-HT, DA, HbA1c, and cholecystokinin—as well as GEM will be recorded at baseline and at the end of the 12-week treatment period (Figure 3).

In addition, the classification of primary and exploratory endpoints was intended to enhance the clarity of outcome evaluation and the interpretability of the study findings. As this was an exploratory trial, the primary purpose of using taVNS was to alleviate gastrointestinal adverse effects associated with semaglutide administration; therefore, metabolic parameters such as fasting insulin, C-peptide, fasting glucagon, fasting plasma glucose, 2-h postprandial glucose, glycated albumin, leptin, adiponectin, lipid profile (triglycerides, total cholesterol, low-density lipoprotein cholesterol, high-density lipoprotein cholesterol), and HbA1c were defined as exploratory endpoints, whereas the RINVR remained the primary endpoint of this study.

#### 2.7.4 Compliance assessment

Participant compliance will be quantitatively assessed using a standardized metric:


$$\text{Compliance Score}\;(\%)=(\begin{array}{l} \text{Number of treatment sessions completed}\\ /\text{Total sessions required} \end{array})\times 100$$

A score ≥80% at Week 12 will define good compliance. For example: a participant completing 6 sessions against the required 12 sessions achieves a score of 50%, indicating inadequate compliance.

#### 2.7.5 Safety and adverse events

Adverse events (AEs) may occur in the taVNS group, including skin redness and swelling, burns, and allergic reactions. If these AEs occur, the investigators will determine whether the events are caused by the treatment. In addition, clinical experts will decide whether to continue or terminate the trial and provide appropriate compensation. AEs induced by taVNS will be recorded in the AE form. These events will be further classified by severity into mild, moderate, and severe adverse events (mild AEs: transient and tolerable events; moderate AEs: events that cause discomfort and interfere with the participant’s normal life; severe AEs: events that seriously affect the participant’s physical health or may pose a risk of death) (39). If AEs occur during treatment, the AE form should be completed, including the time of occurrence, duration, manifestations, measures taken, and outcomes.

In case of intolerance, local bleeding/hematoma, or burns: first, immediately stop the procedure; for participants with intolerance, clarify their tolerance level—if the participant can tolerate and is willing to continue treatment, the intervention plan will be adjusted according to their tolerance and appropriate measures will be taken; for participants with local bleeding/hematoma or burns, the cause should be clarified, the participant should be reassured, and after removing the device, instructed to rest in a supine position with warmth and drink warm water. Meanwhile, the affected area will be disinfected using alcohol or povidone-iodine by medical staff and properly bandaged. If the symptoms do not improve, emergency rescue measures will be taken; finally, it will be determined whether the participant can continue the subsequent treatment. If further treatment is not possible, the study will be terminated. If the participant shows signs of disease aggravation, all trial procedures should be stopped immediately, and symptomatic treatment will be administered by professionals.

### 2.8 Follow-up and data collection

At the end of the trial, all participants will receive a free taVNS device and four health counseling sessions as compensation. Follow-up will be conducted in the outpatient department twelve weeks after the completion of treatment. The assessments will include general information, medication adherence and adjustment, taVNS device usage, and subjective evaluations using RINVR, SNAQ, and VAS scales. In addition, GEM and serological parameters will be measured to evaluate the persistence of therapeutic effects. During the follow-up, investigators will systematically inquire about and document all AEs. If any serious adverse event occurs, an immediate reporting process will be initiated, followed by appropriate medical evaluation and management. For participants lost to follow-up, the reasons will be documented, and efforts will be made to collect available data such as questionnaires through remote means.

### 2.9 Statistic analysis

Exploratory subgroup analyses will be performed to investigate potential differences in taVNS responsiveness according to age, gender, and duration of T2DM. Participants will be stratified into relevant categories (e.g., younger vs. older, male vs. female, shorter vs. longer disease duration), and outcomes will be summarized descriptively within each subgroup. Due to the pilot nature of this study, these analyses are intended for hypothesis generation and will not be formally powered for statistical significance, but they will provide valuable insights to guide the design and sample size calculation of future large-scale trials.

A blinded biostatistician will conduct intention-to-treat (ITT) analyses using SAS (Cary, NC) and R v4.2.1. Baseline characteristics will be summarized by group: continuous variables as mean±SD (normal distribution) or median[IQR] (non-normal), categorical variables as percentages. Primary outcomes (response rates) undergo χ^2^ testing (*α* = 0.025). Secondary outcomes (VAS, SNAQ, GEM, serological markers) will be analyzed via linear mixed-effects models for repeated measures (α = 0.05). All effect sizes will report 95% CIs. Compliance rates and adverse events will be compared using χ^2^ tests. Missing data employs last observation carried forward.

### 2.10 Data management and monitoring

Case report forms (CRFs) will serve as the primary data collection instrument. To safeguard data integrity, routine monitoring and auditing procedures will be implemented. For any participant discontinuing study involvement, withdrawal rationales are required to be documented within the respective CRF. Upon trial completion, investigators shall submit all participant documentation to the research administration at Wangjing Hospital, China Academy of Chinese Medical Sciences. Should over one-quarter of enrolled subjects cease the intervention owing to moderate or severe adverse events (AEs), trial continuity will undergo formal reevaluation.

A dual-entry methodology will be employed for data processing. Within fourteen days following data acquisition, CRF information will be independently input twice by two distinct data entry specialists possessing relevant expertise. Subsequently, these records will be archived in the designated clinical trial database, which guarantees information faithfully represents source documents while fulfilling predefined quality benchmarks.

All investigative and oversight functions will be conducted in strict compliance with Good Clinical Practice (GCP) standards under the institutional oversight of Wangjing Hospital, China Academy of Chinese Medical Sciences. An assigned clinical research associate (CRA) conducts fortnightly site visits during clinical sessions to verify and maintain recorded data quality.

## 3 Discussion

T2DM, as a chronic metabolic disorder, is characterized by insulin resistance and impaired insulin secretion, often accompanied by a range of complications that pose significant threats to patient health. In recent years, semaglutide, a rising star among GLP-1 receptor agonists, has garnered considerable attention in the medical community since its introduction (40). However, due to its direct action on the GI tract and its mechanism of delaying gastric emptying during the first hour postprandially, semaglutide often induces GI side effects such as nausea and vomiting. These adverse effects, particularly pronounced in some patients, can severely compromise treatment adherence and quality of life, thereby limiting its broader clinical adoption and patient acceptance to some extent (1). Consequently, identifying effective strategies to manage and mitigate the GI side effects associated with semaglutide has become a pressing clinical issue.

Common pharmacological agents used to relieve nausea and vomiting—such as ondansetron, metoclopramide, and dexamethasone—are often accompanied by adverse events, including headache, constipation, and hypertension, which may in turn impair treatment compliance. taVNS, a non-invasive neuromodulation technique, modulates visceral function by stimulating specific acupoints within the only surface distribution area of the vagus nerve. In China, taVNS has been increasingly applied to treat common clinical conditions such as GI (20), neurological (41, 42), and endocrine diseases (25). The mechanism by which taVNS may alleviate semaglutide-induced delayed gastric emptying likely involves promoting the secretion of gastrin (43, 44), motilin (45), and cholecystokinin (46–48), as well as modulating the activity of gastric vagal pathways (49, 50). Specifically, gastrin stimulates gastric acid secretion, excites GI smooth muscle, and enhances peristalsis (51); motilin initiates migrating motor complexes (MMCs), which facilitate the clearance of gastric and intestinal contents, thereby promoting gastric emptying (52); cholecystokinin enhances bile secretion, supports gastric emptying, and regulates appetite (53); excitation of gastric vagal nerves promotes acetylcholine release, strengthens gastric smooth muscle contraction, and contributes to both the alleviation of nausea and vomiting and the stimulation of GI hormone release (54). Furthermore, gastrin has been shown to act synergistically with GLP-1 in activating pancreatic *β*-cells for glucose reduction (55), improving β-cell quality (56), and promoting the transdifferentiation of exocrine cells into β-cells (57). cholecystokinin may also improve β-cell survival by modulating apoptosis and mitosis (58), as well as stimulate insulin secretion, thereby contributing to blood glucose regulation. This hypothesis underscores the dual role of taVNS in enhancing semaglutide efficacy and reducing its GI side effects, offering new insight into the management of GLP-1 receptor agonist–related adverse reactions.

Previous clinical studies have not reported severe adverse events associated with taVNS, suggesting a favorable safety profile (39). The current study includes participants aged 18 to 70 years. Particular attention will be given to those aged 60 and above, with strict adherence to inclusion and exclusion criteria during enrollment and close monitoring throughout the trial. Post-trial, stratified analyses will be conducted based on age to evaluate differential treatment responses to taVNS. This study is cautiously designed following relevant guidelines and robust methodological principles, including adequate randomization, allocation concealment, and blinding of participants, outcome assessors, and statisticians, to provide reliable evidence on the efficacy and safety of taVNS in mitigating semaglutide-induced GI side effects and its impact on drug efficacy. Specifically, we will assess gastric myoelectrical activity, the severity of nausea, vomiting, and retching, as well as nutritional and appetite status. Randomization will be computer-generated and group assignments concealed by a third party to minimize selection bias. Participants, outcome assessors, and data analysts will remain blinded to group assignments until statistical analysis is completed. The entire study will be conducted in accordance with traditional Chinese medicine theory and clinical practice norms. Given the absence of existing reports on the effect of taVNS on T2DM, we will also objectively evaluate the synergistic and toxicity-reducing effects of taVNS on semaglutide by measuring metabolic parameters such as blood glucose, blood lipids, and gastric function–related biomarkers.

Nevertheless, several limitations of this study should be acknowledged. First, although sham stimulation was applied to the auricular scapha in the control group—a region primarily innervated by the great auricular and auriculotemporal nerves rather than the auricular branch of the vagus nerve—some degree of placebo response cannot be entirely excluded. Patient expectations, attention to the intervention, and the sensory experience of stimulation may contribute to perceived improvements in GI symptoms, and these nonspecific factors should be taken into account when interpreting the results. We hypothesize that taVNS will exhibit superior therapeutic effects compared to sham-taVNS, and the sham protocol was designed based on validated approaches from previous studies to enhance the scientific rigor of the control condition. Second, this study is a single-center trial with all participants recruited from the same healthcare institution, which may limit the generalizability and external validity of the findings. However, compared with multi-center designs, single-center studies offer higher internal validity by allowing better control of confounding variables. Finally, the relatively small sample size may reduce the power to detect statistically significant differences, increasing the risk of type II error. Nonetheless, as a pilot exploratory trial, the sample size—approximately 30 participants per group—is consistent with recommendations from previous literature and is considered appropriate for evaluating initial feasibility and efficacy trends.

## 4 Conclusion

In China, taVNS is a specialized modality within traditional Chinese medicine. To the best of our knowledge, no clinical studies to date have investigated the potential of taVNS to enhance the efficacy of semaglutide while reducing its GI side effects. This research seeks to provide a low-side-effect, non-pharmacological complementary and alternative treatment approach in clinical settings, with the goal of further improving patient quality of life.

In the future, we will design large-sample, multi-center trials based on the findings of this exploratory study to enhance the generalizability of the results across different populations and healthcare settings. The stimulation parameters will be further optimized, and the inclusion and exclusion criteria will be refined. More objective biomarkers and physiological indicators will be incorporated to ensure a rigorous, comprehensive, and objective evaluation of the therapeutic effects. Moreover, we are committed to improving the functionality of the taVNS device by integrating it with advanced technologies such as artificial intelligence and electroencephalography, enabling more accurate and comprehensive data collection. The intervention duration and follow-up period will also be extended, with increased study visit frequency, to enhance data reliability and sustainability, as well as to facilitate the assessment of delayed-onset adverse events.

If taVNS proves effective in reducing GI side effects and enhancing semaglutide efficacy, it could serve as a novel non-pharmacological adjunct to improve treatment tolerability, adherence, and glycemic control in T2DM. Insights from this pilot trial will guide the design of future full-scale randomized controlled studies, including sample size refinement, optimization of stimulation parameters, outcome selection, and identification of responsive patient subgroups, ultimately supporting evaluation of long-term clinical benefits.

## Glossary

T2DM: Type 2 diabetes mellitus
GI: gastrointestinal
CNS: Central Nervous System
GLP-1: Glucagon-like peptide-1
FAERS: FDA Adverse Event Reporting System
ADEs: adverse drug events
5-HT: Serotonin
DA: Dopamine
taVNS: Transcutaneous auricular vagus nerve stimulation
TCM: Traditional Chinese Medicine
ABVN: auricular branch of the vagus nerve
NTS: nucleus tractus solitarius
DMN: dorsal motor nucleus
FD: functional dyspepsia
IBS-C: irritable bowel syndrome with constipation
SPIRIT: Standard Protocol Items: Recommendations for Interventional Trials
WHO: World Health Organization
FPG: Fasting plasma glucose
2 h-PG: 2-h plasma glucose
HbA1c: Glycated hemoglobin
GA: Glycated albumin
DMC: Data Monitoring Committee
RINVR: Rhodes Index of Nausea, Vomiting and Retching
SNAQ: Simplified nutritional appetite questionnaire
VAS: Visual analogue scale
SM: Serological Markers
GEM: Gastric Electromyography
AEs: Adverse events
PP: per-protocol
CRFs: case report forms
CRA: clinical research associate
MMCs: motilin initiates migrating motor complexes
ITT: Intensive Therapeutic Trial
